# Crystal structure of (*E*)-*N*′-benzyl­idene-1-methyl-4-nitro-1*H*-pyrrole-2-carbohydrazide

**DOI:** 10.1107/S1600536814018054

**Published:** 2014-08-13

**Authors:** Zhijun Wang, Chengyong Zhou, Lei Yan, Jinglin Wang

**Affiliations:** a Department of Chemistry, Changzhi University, Changzhi, Shanxi 046011, People’s Republic of China

**Keywords:** crystal structure, pyrrole-2-carbohydrazide, aroylhydrazones, hydrogen bonding, π–π inter­actions

## Abstract

In the title compound, C_13_H_12_N_4_O_3_, the dihedral angle between the planes of the pyrrole and benzene rings is 7.47 (1)°. In the crystal, mol­ecules are arranged in sheets lying parallel to (101). Neighbouring sheets are linked by N—H⋯O hydrogen bonds, weak π–π [centroid–centroid distance between the pyrrole rings = 3.765 (11) Å] and C—H⋯π inter­actions, forming a three-dimensional structure.

## Related literature   

For applications and structures of aroylhydrazones, see: Krishnamoorthy *et al.* (2012 ▶); Raja *et al.* (2012 ▶); Wang *et al.* (2014 ▶). For similar structures, see: Wang *et al.* (2011 ▶, 2014 ▶). For π–π inter­actions, see: Janiak (2000 ▶).
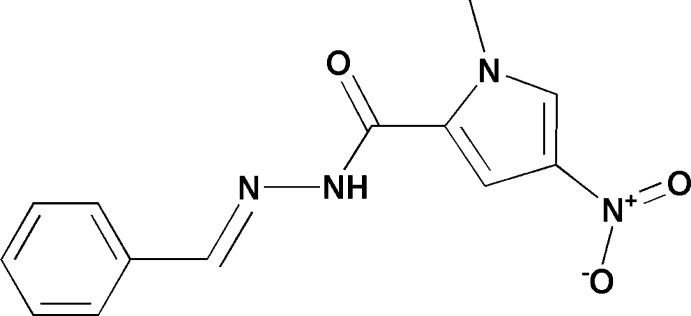


## Experimental   

### Crystal data  

C_13_H_12_N_4_O_3_
*M* *_r_* = 272.27
Monoclinic, 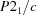

*a* = 13.030 (3) Å
*b* = 11.900 (3) Å
*c* = 8.331 (2) Å
β = 95.409 (3)°
*V* = 1285.92 (17) Å^3^
*Z* = 4
Mo *K*α radiation
μ = 0.10 mm^−1^
*T* = 298 K
0.32 × 0.20 × 0.17 mm

### Data collection  

Bruker SMART 1000 CCD diffractometer
Absorption correction: multi-scan (*SADABS*; Bruker, 2005 ▶) *T*_min_ = 0.968, *T*_max_ = 0.983
6283 measured reflections
2248 independent reflections
1382 reflections with *I* > 2σ(*I*)
*R*_int_ = 0.043

### Refinement  

*R*[*F* ^2^ > 2σ(*F* ^2^)] = 0.047
*wR*(*F* ^2^) = 0.135
*S* = 1.03
2248 reflections
182 parameters
H-atom parameters constrained
Δρ_max_ = 0.19 e Å^−3^
Δρ_min_ = −0.18 e Å^−3^

### 

Data collection: *SMART* (Bruker, 1999 ▶); cell refinement: *SAINT* (Bruker, 1999 ▶); data reduction: *SAINT*; program(s) used to solve structure: *SHELXS97* (Sheldrick, 2008 ▶); program(s) used to refine structure: *SHELXL97* (Sheldrick, 2008 ▶); molecular graphics: *SHELXTL* (Sheldrick, 2008 ▶); software used to prepare material for publication: *SHELXTL* and *DIAMOND* (Brandenburg, 2005 ▶).

## Supplementary Material

Crystal structure: contains datablock(s) I. DOI: 10.1107/S1600536814018054/bt6990sup1.cif

Structure factors: contains datablock(s) I. DOI: 10.1107/S1600536814018054/bt6990Isup2.hkl

Supporting information file. DOI: 10.1107/S1600536814018054/bt6990Isup3.cml

**Fig. 1.**
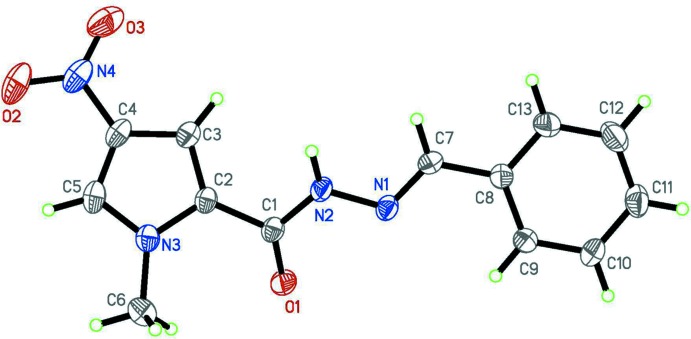
The molecular structure of the title compound with the atom numbering scheme. Displacement ellipsoids are drawn at the 30% probability level. H atoms are represented as small spheres of arbitrary radius.

. DOI: 10.1107/S1600536814018054/bt6990fig1.tif
The mol­ecular structure of the title compound with the atom numbering scheme. Displacement ellipsoids are drawn at the 30% probability level. H atoms are represented as small spheres of arbitrary radius.

**Fig. 2.**
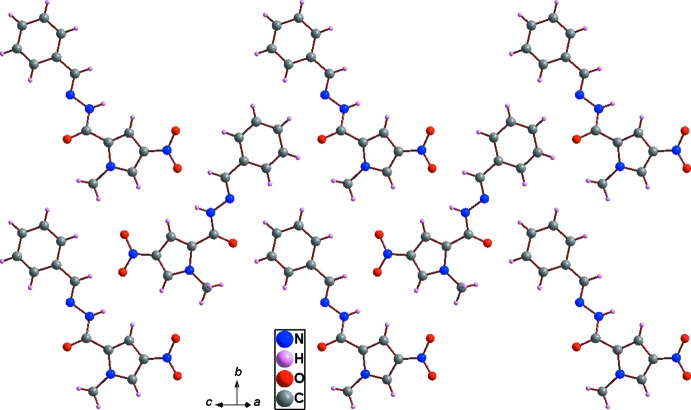
Molecules of the title compound forming planes parallel to the (101) plane.

. DOI: 10.1107/S1600536814018054/bt6990fig2.tif
Mol­ecules of the title compound forming planes parallel to the (101) plane.

**Fig. 3.**
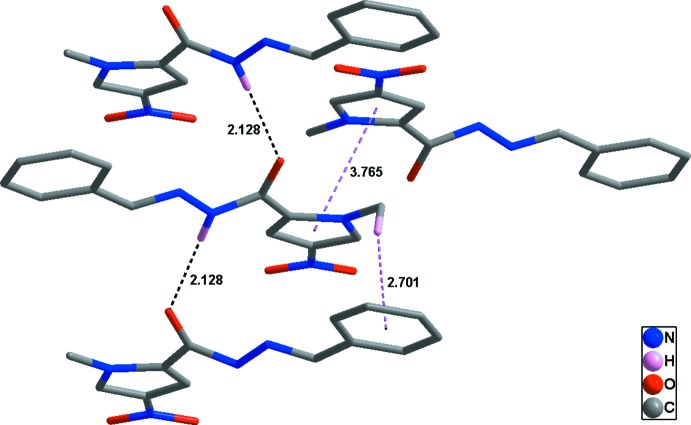
The intermolecular N–H···O hydrogen bonds (black dotted lines), π···π and C–H···π interactions (pink dotted lines) between adjacent sheets (H atoms not involved in hydrogen bonds have been omitted for clarity, all distances in Å).

**Fig. 4.**
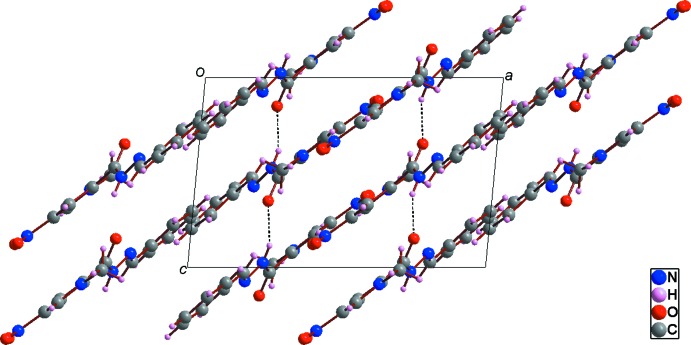
Packing of the title compound viewed along the b axis.

. DOI: 10.1107/S1600536814018054/bt6990fig3.tif
The inter­molecular N–H⋯O hydrogen bonds (black dotted lines), π⋯π and C–H⋯π inter­actions (pink dotted lines) between adjacent sheets (H atoms not involved in hydrogen bonds have been omitted for clarity, all distances in Å).

b . DOI: 10.1107/S1600536814018054/bt6990fig4.tif
Packing of the title compound viewed along the *b* axis.

CCDC reference: 1018158

Additional supporting information:  crystallographic information; 3D view; checkCIF report

## Figures and Tables

**Table 1:** Hydrogen-bond geometry (Å, °) *Cg* is the centroid of the C8–C13 ring.

*D*—H⋯*A*	*D*—H	H⋯*A*	*D*⋯*A*	*D*—H⋯*A*
N2—H2⋯O1^i^	0.86	2.13	2.942 (2)	158
C6—H6*B*⋯*Cg* ^i^	0.96	2.70	3.590 (3)	154

Symmetry code: (i) 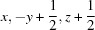.

## supplementary crystallographic information

### S1. Comment

Hydrazones and analogous compounds have attracted attention from researchers due to their well known chelating capability and structural flexibility (Krishnamoorthy, *et al.*, 2012; Raja, *et al.*, 2012). In our lab, a series of asymmetric N-heterocyclic substituted hydrazones and their metal complexes were obtained and characterized (Wang *et al.*, 2011). The interactions of these compounds with CT-DNA and pBR322 DNA has been explored (Wang *et al.*, 2014). The present work is an extension of our earlier studies.

In the title compound (Fig. 1) the phenyl and pyrrolyl ring are linked by an acyl-hydrazone moiety. The dihedral angle between the phenyl and pyrrolyl rings is 7.47 (1)°.

As shown in Figure 2, molecules of the title compound form sheet parallel to the (101) plane.

The neighbouring sheets are linked by N–H···O hydrogen bonds, weak π···π interactions between pyrrolyl rings and C–H···π interactions (Figure 3). These interactions result in the formation of a three-dimensional network (Fig. 4).

### S2. Experimental

The precursor 1-methyl-4-nitro-1*H*-pyrrole-2-carbohydrazide was synthesized according to literature procedures (Wang *et al.*, 2011). Similar to the synthesis of (*E*)-1-methyl-4-nitro-*N'*-(pyridin-2-ylmethylene)-1*H*-pyrrole-2-carbohydrazide, the reaction of the precursor and benzaldehyde in a 1:1 molar ratio gave the title compound as a yellowish powder in 80% yield. Anal. Calc. (%) for C_13_H_12_N_4_O_3_: C 57.35, H 4.44, N 20.58; found: C 57.31, H 4.51, N 20.55. The powder of the title compound was dissolved in *N*,*N*-dimethyl formamide and the yellow crystals were collected after slow evaporation at room temperature for about two weeks.

### S3. Refinement

H atoms were placed in geometrically idealized positions, with N–H=0.86 Å, C_aromatic_–H=0.93, C_methyl_–H 0.96 Å, and with *U*_iso_(H) = *k* × *U*_eq_(parent C-atom), where *k* = 1.5 for methyl H-atoms and 1.2 for other H-atoms.

### Crystal data

**Table:**

C_13_H_12_N_4_O_3_	*F*(000) = 568
*M**_r_* = 272.27	*D*_x_ = 1.406 Mg m^−^^3^
Monoclinic, *P*2_1_/*c*	Mo *K*α radiation, λ = 0.71073 Å
Hall symbol: -P 2ybc	Cell parameters from 1529 reflections
*a* = 13.030 (3) Å	θ = 2.3–24.0°
*b* = 11.900 (3) Å	µ = 0.10 mm^−^^1^
*c* = 8.331 (2) Å	*T* = 298 K
β = 95.409 (3)°	Block, yellow
*V* = 1285.92 (17) Å^3^	0.32 × 0.20 × 0.17 mm
*Z* = 4	

### Data collection

**Table:**

Bruker SMART 1000 CCD diffractometer	2248 independent reflections
Radiation source: fine-focus sealed tube	1382 reflections with *I* > 2σ(*I*)
Graphite monochromator	*R*_int_ = 0.043
phi and ω scans	θ_max_ = 25.0°, θ_min_ = 2.3°
Absorption correction: multi-scan (*SADABS*; Bruker, 2005)	*h* = −13→15
*T*_min_ = 0.968, *T*_max_ = 0.983	*k* = −14→14
6283 measured reflections	*l* = −9→9

### Refinement

**Table:**

Refinement on *F*^2^	Primary atom site location: structure-invariant direct methods
Least-squares matrix: full	Secondary atom site location: difference Fourier map
*R*[*F*^2^ > 2σ(*F*^2^)] = 0.047	Hydrogen site location: inferred from neighbouring sites
*wR*(*F*^2^) = 0.135	H-atom parameters constrained
*S* = 1.03	*w* = 1/[σ^2^(*F*_o_^2^) + (0.0571*P*)^2^ + 0.2921*P*] where *P* = (*F*_o_^2^ + 2*F*_c_^2^)/3
2248 reflections	(Δ/σ)_max_ < 0.001
182 parameters	Δρ_max_ = 0.19 e Å^−^^3^
0 restraints	Δρ_min_ = −0.18 e Å^−^^3^

### Special details

**Table:**

Geometry. All e.s.d.'s (except the e.s.d. in the dihedral angle between two l.s. planes) are estimated using the full covariance matrix. The cell e.s.d.'s are taken into account individually in the estimation of e.s.d.'s in distances, angles and torsion angles; correlations between e.s.d.'s in cell parameters are only used when they are defined by crystal symmetry. An approximate (isotropic) treatment of cell e.s.d.'s is used for estimating e.s.d.'s involving l.s. planes.
Refinement. Refinement of *F*^2^ against ALL reflections. The weighted *R*-factor *wR* and goodness of fit *S* are based on *F*^2^, conventional *R*-factors *R* are based on *F*, with *F* set to zero for negative *F*^2^. The threshold expression of *F*^2^ > σ(*F*^2^) is used only for calculating *R*-factors(gt) *etc*. and is not relevant to the choice of reflections for refinement. *R*-factors based on *F*^2^ are statistically about twice as large as those based on *F*, and *R*- factors based on ALL data will be even larger.

### Fractional atomic coordinates and isotropic or equivalent isotropic displacement parameters (Å^2^)

**Table:**

	*x*	*y*	*z*	*U*_iso_*/*U*_eq_	
N1	0.80415 (15)	0.34342 (17)	0.4275 (2)	0.0409 (5)	
N2	0.74919 (15)	0.27603 (17)	0.5233 (2)	0.0435 (6)	
H2	0.7314	0.3006	0.6138	0.052*	
N3	0.64881 (15)	−0.00154 (17)	0.5892 (2)	0.0436 (6)	
N4	0.45223 (18)	0.0771 (3)	0.8358 (3)	0.0618 (7)	
O1	0.75036 (14)	0.12934 (15)	0.3497 (2)	0.0540 (5)	
O2	0.41349 (15)	−0.0109 (2)	0.8783 (2)	0.0804 (7)	
O3	0.42286 (18)	0.1703 (2)	0.8735 (3)	0.0916 (8)	
C1	0.72375 (18)	0.1713 (2)	0.4734 (3)	0.0397 (6)	
C2	0.65549 (18)	0.1145 (2)	0.5783 (3)	0.0386 (6)	
C3	0.58369 (18)	0.1601 (2)	0.6690 (3)	0.0441 (7)	
H3	0.5704	0.2361	0.6829	0.053*	
C4	0.53470 (19)	0.0704 (2)	0.7359 (3)	0.0469 (7)	
C5	0.57607 (19)	−0.0277 (2)	0.6867 (3)	0.0502 (7)	
H5	0.5572	−0.0998	0.7156	0.060*	
C6	0.7129 (2)	−0.0846 (2)	0.5155 (3)	0.0560 (8)	
H6A	0.6865	−0.1586	0.5319	0.084*	
H6B	0.7825	−0.0796	0.5644	0.084*	
H6C	0.7117	−0.0700	0.4020	0.084*	
C7	0.81171 (19)	0.4454 (2)	0.4711 (3)	0.0419 (6)	
H7	0.7786	0.4697	0.5589	0.050*	
C8	0.87158 (18)	0.5250 (2)	0.3853 (3)	0.0388 (6)	
C9	0.93840 (19)	0.4903 (2)	0.2762 (3)	0.0467 (7)	
H9	0.9426	0.4143	0.2512	0.056*	
C10	0.9988 (2)	0.5662 (2)	0.2040 (3)	0.0530 (8)	
H10	1.0445	0.5413	0.1324	0.064*	
C11	0.9919 (2)	0.6776 (3)	0.2370 (3)	0.0604 (8)	
H11	1.0330	0.7290	0.1885	0.072*	
C12	0.9246 (3)	0.7139 (2)	0.3416 (4)	0.0724 (10)	
H12	0.9186	0.7903	0.3620	0.087*	
C13	0.8655 (2)	0.6380 (2)	0.4170 (3)	0.0587 (8)	
H13	0.8210	0.6635	0.4901	0.070*	

### Atomic displacement parameters (Å^2^)

**Table:**

	*U*^11^	*U*^22^	*U*^33^	*U*^12^	*U*^13^	*U*^23^
N1	0.0444 (12)	0.0449 (13)	0.0346 (12)	−0.0062 (10)	0.0097 (10)	0.0028 (10)
N2	0.0533 (13)	0.0436 (12)	0.0362 (13)	−0.0105 (10)	0.0172 (10)	−0.0027 (10)
N3	0.0437 (13)	0.0442 (13)	0.0428 (13)	−0.0056 (10)	0.0024 (10)	0.0019 (11)
N4	0.0421 (14)	0.096 (2)	0.0474 (15)	−0.0117 (15)	0.0069 (12)	0.0073 (16)
O1	0.0735 (13)	0.0526 (11)	0.0388 (11)	−0.0083 (10)	0.0211 (10)	−0.0081 (9)
O2	0.0547 (13)	0.1207 (19)	0.0668 (15)	−0.0377 (13)	0.0107 (11)	0.0153 (14)
O3	0.0764 (17)	0.112 (2)	0.0932 (19)	0.0082 (15)	0.0433 (14)	−0.0026 (17)
C1	0.0425 (15)	0.0449 (16)	0.0319 (15)	−0.0037 (12)	0.0039 (12)	0.0031 (13)
C2	0.0400 (14)	0.0439 (15)	0.0316 (14)	−0.0057 (12)	0.0009 (11)	0.0038 (12)
C3	0.0436 (15)	0.0507 (16)	0.0385 (16)	−0.0035 (13)	0.0065 (13)	0.0029 (13)
C4	0.0354 (14)	0.0679 (19)	0.0379 (16)	−0.0091 (14)	0.0054 (12)	0.0040 (14)
C5	0.0473 (17)	0.0566 (18)	0.0455 (17)	−0.0171 (14)	−0.0013 (14)	0.0101 (14)
C6	0.0604 (19)	0.0455 (16)	0.061 (2)	0.0004 (14)	0.0017 (15)	−0.0036 (14)
C7	0.0439 (15)	0.0466 (16)	0.0367 (15)	0.0009 (13)	0.0115 (12)	−0.0018 (13)
C8	0.0417 (14)	0.0402 (15)	0.0343 (15)	−0.0041 (11)	0.0023 (12)	0.0002 (12)
C9	0.0474 (16)	0.0388 (15)	0.0552 (17)	0.0036 (12)	0.0116 (14)	0.0038 (13)
C10	0.0493 (16)	0.0574 (19)	0.0547 (19)	−0.0070 (14)	0.0177 (14)	0.0038 (15)
C11	0.075 (2)	0.0553 (19)	0.0521 (19)	−0.0224 (16)	0.0116 (16)	0.0043 (15)
C12	0.110 (3)	0.0413 (17)	0.069 (2)	−0.0147 (18)	0.025 (2)	−0.0071 (16)
C13	0.078 (2)	0.0486 (18)	0.0523 (19)	−0.0055 (15)	0.0238 (16)	−0.0101 (15)

### Geometric parameters (Å, º)

**Table:**

N1—C7	1.268 (3)	C6—H6A	0.9600
N1—N2	1.379 (3)	C6—H6B	0.9600
N2—C1	1.346 (3)	C6—H6C	0.9600
N2—H2	0.8600	C7—C8	1.456 (3)
N3—C5	1.342 (3)	C7—H7	0.9300
N3—C2	1.386 (3)	C8—C13	1.374 (3)
N3—C6	1.466 (3)	C8—C9	1.380 (3)
N4—O3	1.224 (3)	C9—C10	1.374 (3)
N4—O2	1.230 (3)	C9—H9	0.9300
N4—C4	1.422 (3)	C10—C11	1.359 (4)
O1—C1	1.224 (3)	C10—H10	0.9300
C1—C2	1.469 (3)	C11—C12	1.363 (4)
C2—C3	1.369 (3)	C11—H11	0.9300
C3—C4	1.387 (3)	C12—C13	1.376 (4)
C3—H3	0.9300	C12—H12	0.9300
C4—C5	1.365 (4)	C13—H13	0.9300
C5—H5	0.9300		
			
C7—N1—N2	114.9 (2)	N3—C6—H6B	109.5
C1—N2—N1	119.1 (2)	H6A—C6—H6B	109.5
C1—N2—H2	120.5	N3—C6—H6C	109.5
N1—N2—H2	120.5	H6A—C6—H6C	109.5
C5—N3—C2	108.8 (2)	H6B—C6—H6C	109.5
C5—N3—C6	124.1 (2)	N1—C7—C8	120.8 (2)
C2—N3—C6	127.0 (2)	N1—C7—H7	119.6
O3—N4—O2	123.5 (3)	C8—C7—H7	119.6
O3—N4—C4	118.2 (3)	C13—C8—C9	118.1 (2)
O2—N4—C4	118.3 (3)	C13—C8—C7	119.9 (2)
O1—C1—N2	123.8 (2)	C9—C8—C7	121.9 (2)
O1—C1—C2	123.3 (2)	C10—C9—C8	121.0 (2)
N2—C1—C2	112.8 (2)	C10—C9—H9	119.5
C3—C2—N3	108.0 (2)	C8—C9—H9	119.5
C3—C2—C1	129.0 (2)	C11—C10—C9	120.1 (3)
N3—C2—C1	122.8 (2)	C11—C10—H10	119.9
C2—C3—C4	106.3 (2)	C9—C10—H10	119.9
C2—C3—H3	126.8	C10—C11—C12	119.8 (3)
C4—C3—H3	126.8	C10—C11—H11	120.1
C5—C4—C3	109.1 (2)	C12—C11—H11	120.1
C5—C4—N4	124.4 (3)	C11—C12—C13	120.4 (3)
C3—C4—N4	126.4 (3)	C11—C12—H12	119.8
N3—C5—C4	107.8 (2)	C13—C12—H12	119.8
N3—C5—H5	126.1	C8—C13—C12	120.6 (3)
C4—C5—H5	126.1	C8—C13—H13	119.7
N3—C6—H6A	109.5	C12—C13—H13	119.7
			
C7—N1—N2—C1	170.0 (2)	O3—N4—C4—C3	−3.2 (4)
N1—N2—C1—O1	3.3 (4)	O2—N4—C4—C3	176.1 (3)
N1—N2—C1—C2	−173.4 (2)	C2—N3—C5—C4	1.1 (3)
C5—N3—C2—C3	−1.1 (3)	C6—N3—C5—C4	178.1 (2)
C6—N3—C2—C3	−178.0 (2)	C3—C4—C5—N3	−0.7 (3)
C5—N3—C2—C1	−176.4 (2)	N4—C4—C5—N3	177.4 (2)
C6—N3—C2—C1	6.7 (4)	N2—N1—C7—C8	177.2 (2)
O1—C1—C2—C3	−146.8 (3)	N1—C7—C8—C13	169.5 (3)
N2—C1—C2—C3	29.9 (4)	N1—C7—C8—C9	−13.1 (4)
O1—C1—C2—N3	27.4 (4)	C13—C8—C9—C10	1.4 (4)
N2—C1—C2—N3	−155.9 (2)	C7—C8—C9—C10	−176.0 (2)
N3—C2—C3—C4	0.7 (3)	C8—C9—C10—C11	−1.3 (4)
C1—C2—C3—C4	175.6 (2)	C9—C10—C11—C12	−0.2 (5)
C2—C3—C4—C5	0.0 (3)	C10—C11—C12—C13	1.7 (5)
C2—C3—C4—N4	−178.1 (2)	C9—C8—C13—C12	0.0 (4)
O3—N4—C4—C5	179.0 (3)	C7—C8—C13—C12	177.5 (3)
O2—N4—C4—C5	−1.7 (4)	C11—C12—C13—C8	−1.6 (5)

### Hydrogen-bond geometry (Å, º)

*Cg* is the centroid of the C8–C13 ring.

**Table:**

*D*—H···*A*	*D*—H	H···*A*	*D*···*A*	*D*—H···*A*
N2—H2···O1^i^	0.86	2.13	2.942 (2)	158
C6—H6*B*···*Cg*^i^	0.96	2.70	3.590 (3)	154

Symmetry code: (i) *x*, −*y*+1/2, *z*+1/2.
