# Supplementary figures and images for: Crystal structure of (E)-N′-benzyl­idene-1-methyl-4-nitro-1H-pyrrole-2-carbohydrazide
Source: Acta Crystallogr Sect E Struct Rep Online. 2014 Aug 13;70(Pt 9):o995. doi: 10.1107/S1600536814018054 (PMC4186152; doi:10.1107/S1600536814018054)

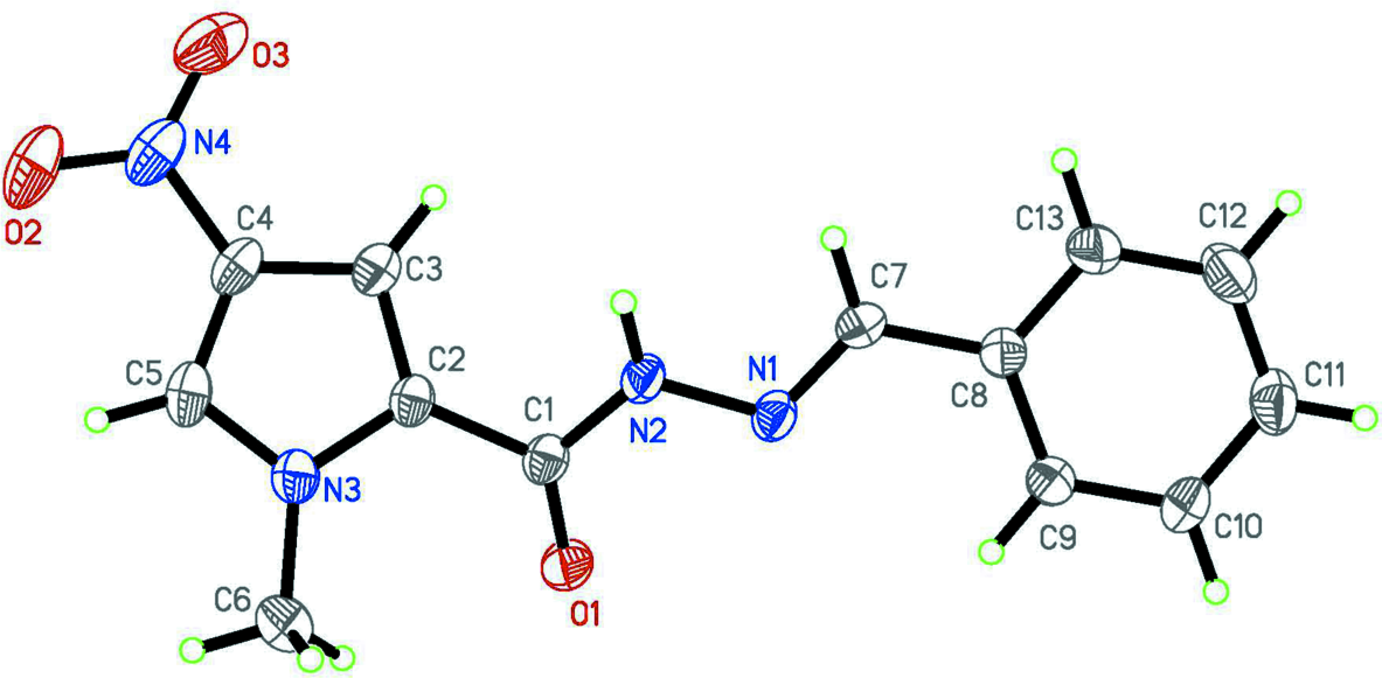

Supplement: Supplementary file 4 [file e-70-0o995-fig1.tif]

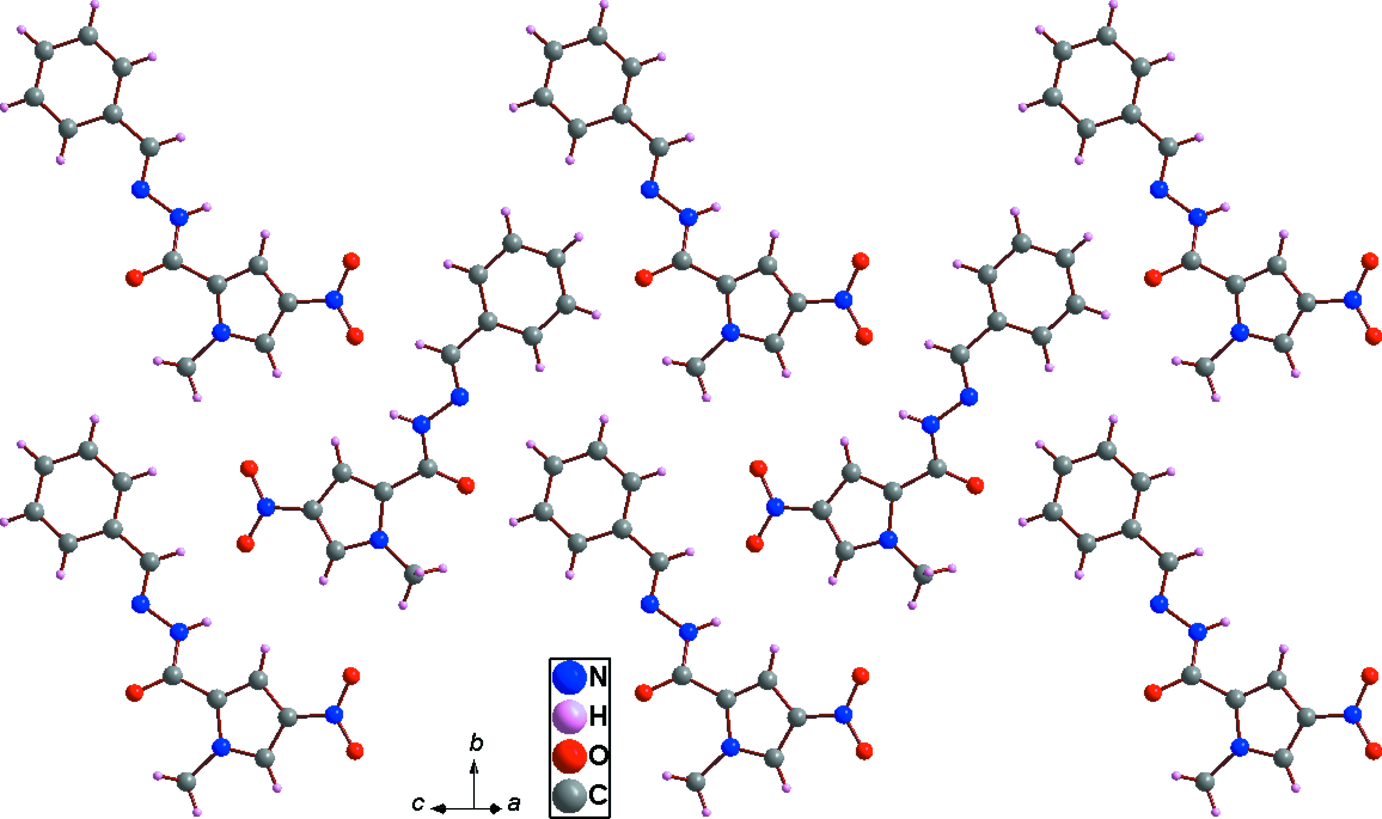

Supplement: Supplementary file 5 [file e-70-0o995-fig2.tif]

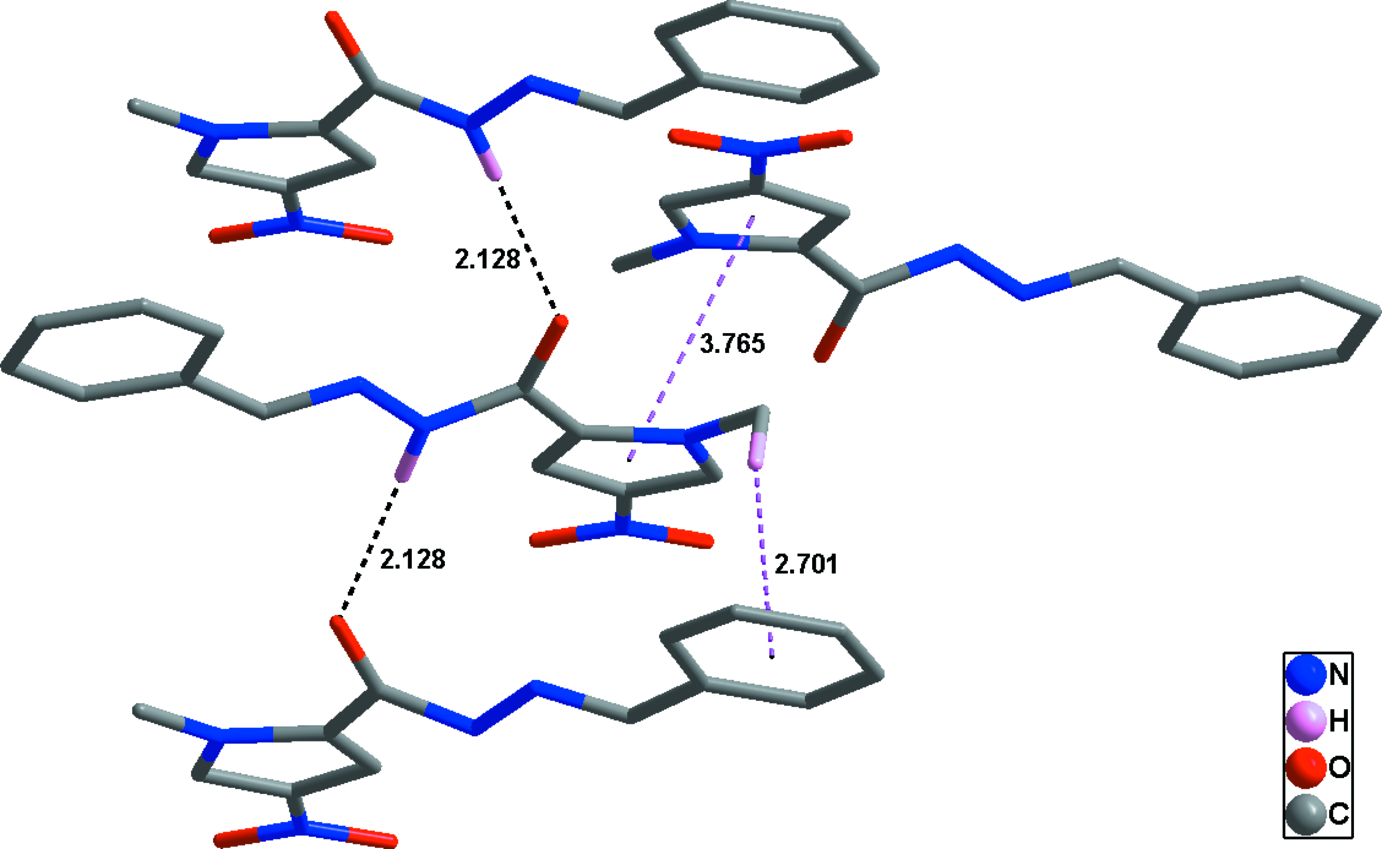

Supplement: Supplementary file 6 [file e-70-0o995-fig3.tif]

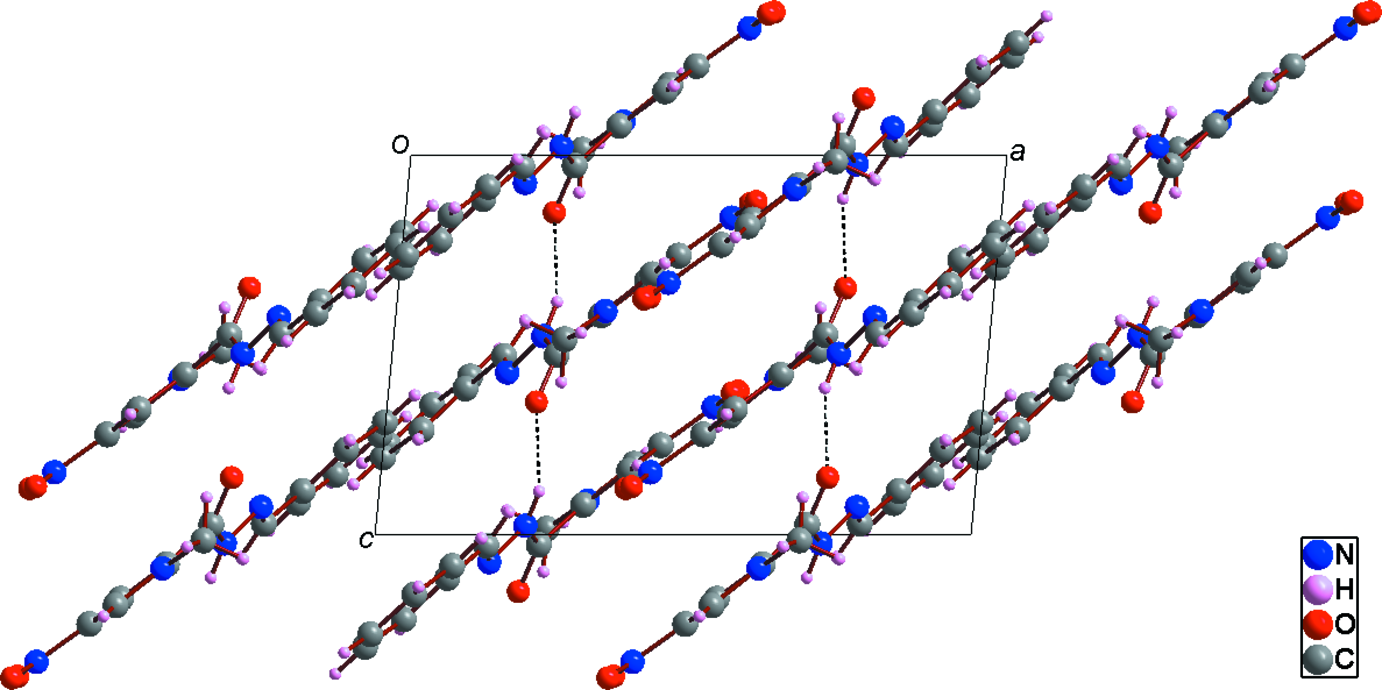

Supplement: Supplementary file 7 [file e-70-0o995-fig4.tif]
